# ERK/mTOR signaling may underlying the antidepressant actions of rapastinel in mice

**DOI:** 10.1038/s41398-022-02290-5

**Published:** 2022-12-22

**Authors:** Mengxin Shen, Dan Lv, Xinya Liu, Chuang Wang

**Affiliations:** 1 Zhejiang Key Laboratory of Pathophysiology, School of Medicine, Ningbo University, 315211 Ningbo, Zhejiang People’s Republic of China; 2 School of Basic Medical Science, School of Medicine, Ningbo University, 315211 Ningbo, Zhejiang People’s Republic of China; 3 Central Laboratory, Ningbo First Hospital, Zhejiang University, 315010 Ningbo, Zhejiang China

**Keywords:** Depression, Clinical pharmacology, Molecular neuroscience

## Abstract

Rapastinel as the allosteric modulator of N-methyl-D-aspartate receptor (NMDAR) produces rapid antidepressant-like effects dependent on brain-derived neurotrophic factor (BDNF) and VGF (nonacryonimic) release. Herein, we further explore the molecular mechanisms of the antidepressant effects of repeated administration with rapastinel in mice. Our results showed that continuous 3-day rapastinel (5 and 10 mg/kg, i.v.) produced antidepressant-like actions dependent on the increase in extracellular regulated protein kinase (ERK)/mammalian target of rapamycin (mTOR) signaling and downstream substrates p70S6 kinase (p70S6k) and the eukaryotic initiation factor 4E-binding protein 1 (4E-BP1), which may induce the expression of VGF and BDNF in the hippocampus and prefrontal cortex of mice. Furthermore, compared with a single treatment, our data indicated that 3-day repeated rapastinel treatment produced antidepressant-like actions accompanied by potentiation of ERK/mTOR/VGF/BDNF/tropomyosin-related kinase receptor B (TrkB) signaling. Based on previous and our supplementary data that showed the pivotal role of on α-amino-3-hydroxy-5-methyl-4-isoxazolepropionic acid receptor (AMPAR) in the rapid release of VGF and BDNF and activation of TrkB by a single dose of rapastinel, we postulate that the antidepressant-like effects of single or repeated administration of rapastinel may result in the rapid release of VGF and BDNF or ERK/mTOR signaling pathway-mediated VGF/BDNF/TrkB autoregulatory feedback loop respectively. Our current work adds new knowledge to the molecular mechanisms that underlie the antidepressant-like actions of rapastinel in mice.

## Introduction

Major depressive disorder (MDD) is a chronic and devastating mental disorder with a high prevalence and incidence [1]. Delayed onset of antidepressant action is a major limitation of all existing conventional antidepressant therapies: typically, 3–8 weeks of repeated treatment is required to produce antidepressant-like effects [2–4]. Additionally, approximately one-third of patients with depression fail to respond to conventional antidepressants [5]. Furthermore, these limitations prolong depression, disability, and suicide risk in patients with MDD. Therefore, the development of fast and efficient antidepressants without these limitations remains a major challenge.

Previous research had indicated that ketamine has a rapid antidepressant effect in patients with drug-resistant MDD [6–9]. Ketamine is an N-methyl-D-aspartate receptor (NMDAR) antagonist that can promote the synthesis and transmission of glutamate, activate brain-derived neurotrophic factor (BDNF)/tropomyosin-related kinase receptor B (TrkB) signal transduction, and promote synaptic formation, thus achieving a rapid and enduring antidepressant effect [10, 11]. However, given the limitations of psychotomimetic effects, its long-term use is restricted due to its side effects. Therefore, there is an urgent need for antidepressants with rapid and lasting effects, similar to ketamine, without its limitations.

It used to be thought that rapastinel (formerly GLYX-13) is a novel glutamatergic compound that acts as an NMDAR modulator with glycine-like partial agonist properties [12–14], like the NMDAR antagonist ketamine produces rapid antidepressant actions and without the side effects seen with ketamine.In contrast, recently study [15] revealed that rapastinel at therapeutic concentrations directly enhances NMDAR through a novel site independent of the glycine co-agonist site, demonstrating that it is a co-agonist with glutamate rather than a functional glycine site partial agonist as previously thought. This results demonstrated that rapastinel is a weak modulator that enhances NMDAR activity at antidepressant-like concentrations and inhibits NMDAR activity at higher concentrations [15], indicating that rapastinel as an allosteric modulator of NMDAR. Although rapastinel and ketamine physically target NMDAR, the two molecules have opposing actions on NMDAR. The allosteric modulation of NMDAR by rapastinel represents a novel pharmacological approach to promote well-tolerated, rapid, and sustained improvements in depression [15]. A single administration of rapatinel has produced rapid antidepressant actions in clinical phase II [14] and preclinical rodent models [13, 16–18], without psychotomimetic or dissociative effects [19]. In addition to the antidepressant-like effects of rapastinel, improvements in anxiety, cognitive impairment, cerebral ischemia injury, post-traumatic stress disorder, autism, schizophrenia, and obsessive-compulsive disorder have been widely reported [20–28]. However, four recent phase III studies (ClinicalTrials.gov Identifiers NCT02932943, NCT02943564, NCT02943577, NCT02951988) on the use of rapastinel as an adjunctive compound for MDD did not identify rapid-acting antidepressant-like actions. The influence of complex panels (e.g., heterogeneity of MDD, diagnostic criteria, injection scheme, and placebo response) in clinical trials may explain the failure of clinical trials of rapastinel.

An increasing number of studies have demonstrated that BDNF and VGF (nonacryonimic) proteins are decreased in animal models of depression [10, 29–32]. Mounting evidence has indicated that rapid release of BDNF and VGF plays a critical role in rapid-acting antidepressants (e.g., ketamine, rapastinel, and scopolamine) [30, 33, 34]. Notably, these rapid-acting antidepressant compounds stimulate BDNF receptor tropomyosin-related kinase receptor B (TrkB) and activate the mammalian target of rapamycin (mTOR) pathway and its downstream substrates, such as ribosomal protein S6 kinase (P70S6K) and eukaryotic initiation factor 4E-binding protein 1 (4E-BP1), which subsequently lead to convergent effects [35] and protein translation [36, 37]. In addition, further research revealed that TrkB activates the downstream of AKT, also named protein kinase B (PKB) and extracellular regulated protein kinase (ERK) mediated mTOR signaling, suggesting that the phosphorylation of ERK and AKT and activation of the common downstream molecular mTOR participate in the antidepressant-like effects of rapastinel in vivo [38, 39]. Previous reports focused on the AKT/mTOR signaling in rapid-acting actions of rapastinel; [18] however, it remains unclear whether ERK/mTOR signaling is key in the rapid-acting antidepressant-like effects of repeated rapastinel treatment in vivo. Herein, repeated or single treatment of rapastinel was used to better understand whether the ERK/mTOR-mediated VGF/BDNF/TrkB autoregulator feedback loop played a role in the antidepressant-like actions of rapastinel in preclinical models.

## Materials and methods

### Animals

Adult male C57BL/6J mice (weight, 20–25 g on the first day of experiment) were reared in the animal facility of Ningbo University School of Medicine, China. All animals were maintained at 22 °C ± 2 °C and 60% ± 5% relative humidity under a 12-h-light/-dark cycle (lights on at 7:00 am) with ad libitum access to food and water when the stressors were not applied. Required animals were estimated based on our past experience of performing similar experiments and animals were randomly assigned to treatment groups. All stressors were applied to animals outside their housing areas in a separate procedure room. All procedures involving animals were conducted in accordance with the National Institute of Health Guidelines for the Care and Use of Laboratory Animals (NIH Publications No. 80-23, revised 1996) and the European Community Council Directive for the Care and Use of Laboratory Animals of September 22, 2010 (2010/63/EU). All experiments were approved by the Institutional Animal Care and Use Committee of Ningbo University, School of Medicine.

### Drugs and stereotaxic intracranial injections

The following drugs were used: rapastinel (0.5, 5, and 10 mg/kg, i.v., Tocris Bioscience, Cat# 3406) and PD98059 (5 nmol/mice, i.c.v., Abcam, USA, Cat#ab120234). The doses of rapastinel were based on previously reported clinical and preclinical studies [13, 14] and were confirmed in our preliminary experiment. These solutions were dissolved in 0.9% saline, freshly prepared before administration. Mice were anesthetized in an induction box with 3.5% isoflurane and subsequently maintained with 2.5% isoflurane through a nose cone and placed in a stereotaxic frame. The infusion cannula was implanted bilaterally into the intracerebroventricular (i.c.v.) space according the following bregma coordinated: −1.0 mm lateral, ±0.3 mm posterior, and −2.5 mm below. The 10-µl Hamilton microsyringe with a 30-gauge needle was used in the bilateral i.c.v. infusions. Animals were allowed recovery for 7 days.

**Fig. 1:**
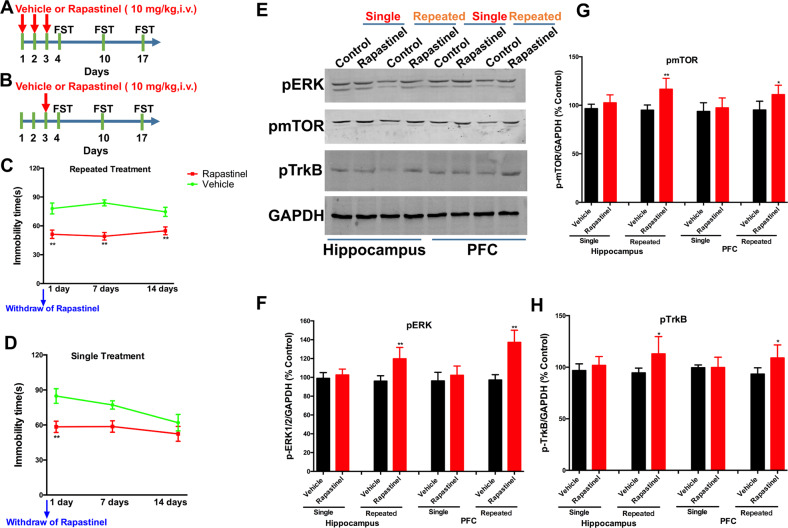
The antidepressant-like effects of repeated or single treatment with rapastinel and changes in the expression of p-ERK, p-mTOR, and p-TrkB in mice. **A** and **B** Experimental timeline schematic. **C** Repeated treatment with rapastinel significantly decreased the immobility time at 1, 7, and 14 days after withdraw of rapastinel. **D** However, single treatment with rapastinel significantly produced significant inhibition in immobility time only at 1 day after withdrawal of rapastinel. Our data indicated that repeated treatment with rapastinel induced the significant antidepressant-like effects in the FST of mice. However, single treatment with rapastinel induced antidepressant-like effects that only lasted a week. Based on our hypothesis about the VGF/BDNF/TrkB autoregulatory feedback loop and increase of ERK/mTOR signaling that may induce the significantly antidepressant-like actions, we performed western blotting to detect the levels of p-ERK, p-mTOR, and p-TrkB in the hippocampus and PFC of mice (**E**). Repeated treatment with rapastinel showed a significant increase in p-ERK (**F**), p-mTOR (**G**), and pTrkB (**H**) both in the hippocampus and PFC of mice after 14 days of withdrawal of rapastinel. However, the levels of these proteins were not changed by single treatment with rapastinel in the hippocampus and PFC of mice after 14 days of withdrawal of rapastinel. Data are expressed as means ± S.E.M; *n* = 10 per group for behavioral tasks and *n* = 5 per group for western blotting tests. *P < 0.05, **P < 0.01 compared with the vehicle group.

The first set of experiments were performed to compare the differences between repeated and single treatment with rapastinel in mice (the timeline has been shown in Fig. 1A, B). The second experiment was to investigate the rapid antidepressant-like effect of different concentrations of rapastinel in a chronic unpredictable stress (CUS) paradigm-induced mouse model and explore whether the rapid antidepressant-like effect is related to ERK/mTOR/BDNF/VGF signaling in the prefrontal cortex (PFC) of mice. Rapastinel (0.5, 5, and 10 mg/kg, i.v.) or vehicle (i.v.) were administered to the mice. The behavioral tasks were conducted in the light phase between 8:00 a.m. and 4:00 p.m. separately on days 36, 37, 38, 39, 40, and 41 (the timeline has been shown in Fig. 3A). The third set of experiments were performed to confirm whether the inhibition of ERK blocked the rapid antidepressant-like effects and upregulation of ERK/mTOR/BDNF/VGF signaling by rapastinel in mice. The ERK antagonist PD98059 (5 nmol/mice) was infused into the bilateral PFC at a rate of 0.5 µl/min 1 h before CUS, and tail vein injection of rapastinel 30 min later for 3 days. The mice were then subjected to the behavioral tasks successively from day 36 to day 41 (the timeline has been shown in Fig. 4A). Analyses were performed in a manner blinded to treatment assignments in all behavioral experiments.

### Chronic unpredictable stress procedure

Eleven different stressors in a random order were used during the 5-week period (as shown in the supplementary data Table S1).

### Open-field test (OFT)

The OFT is commonly used to assess locomotor activity in rodents. We used a white square Plexiglas box (50 × 50 × 40 cm) that was divided by two cross black lines drawn on the floor. Mice were placed in the arena and allowed to explore for 5 min. The number of the line crossings and rearings were considered as parameters of locomotor activity and were recorded over the 5-min period by a digital system.

### Sucrose preference test (SPT)

SPT is a method to evaluate anhedonic-like behavior in animals based on sucrose taste preference. All mice were trained to adapt to the two bottles, one containing 1% sucrose solution and the other with fresh water for 24 h. The positions of the two bottles were randomly placed and were exchanged to prevent the formation of position preference. After adaptation, the mice were deprived of water for 24 h. The mice were housed in individual cages and had free access to two bottles containing sucrose solution and water. After 16 h, the volumes of the sucrose and water were measured and the sucrose preference rate was calculated as a percentage of the amount of sucrose intake/(water intake + sucrose intake) × 100%.

### Novelty suppressed feeding test (NSFT)

NSFT was conducted over a 6-min period. Briefly, mice were food deprived for 24 h and then placed in a novel environment (50 × 50 × 40 cm Plexiglas chamber). The latency to approach and feed on a single food pellet (defined as the mouse chewing or biting the pellet, but not merely sniffing or toying with the pellet) placed on a white paper platform positioned at the center of the brightly lit box was measured.

### Forced swimming test (FST)

The mice were placed individually for 6 min into a clear plastic cylinder (diameter, 10 cm; height, 25 cm) filled with 10-cm depth of water (maintained at 23 ± 2 °C). The immobility time was recorded over the following 4 min of the 6-min testing duration. The immobility time was defined as time when mouse floated with only active activity necessary to keep their heads above the water.

After the last behavioral task, the animals were immediately sacrificed for biochemical assays.

### Western blot analysis

Frozen hippocampal and PFC tissues in each group of mice were homogenized in ice-cold radioimmunoprecipitation assay lysis (RIPA) buffer containing protease and phosphatase inhibitor cocktail (Pierce Biotechnology, Rockford, IL, USA, Cat#89900). Lysates were centrifuged at 12,000 × *g* for 30 min at 4 °C. The protein concentration of each sample lysate was determined using a BCA kit (Thermo Scientific, Rockford, IL, USA, Cat#23225). Each sample (25 µg total protein) was separated on 10% SDS polyacrylamide gel electrophoresis gels and transferred to PVDF membranes (0.22 µm; Millipore, CA, Cat#SLGVR13SL). The membranes were then incubated with anti-phospho-ERK1/2 (Thr204/187) (1:1000, Sigma-Aldrich, USA, Cat#:E7028), anti-ERK1/2 (1:1000, Cell Signaling, USA, Cat#9101), anti-phospho-mTOR (Ser2448) (1:1000, Cell Signaling, USA, Cat#2971), anti-phospho-p70S6K (Thr389) (1:1000, Cell Signaling, USA, Cat#9205), anti-phospho-4E-BP1 (Thr37/46) (1:1000, Cell Signaling, USA, Cat#9459), and anti-GAPDH (1:2000, Millipore, CA, USA, Cat#AB2302) at 4 °C overnight. The membranes were then incubated with an Alexa Fluor 800-conjugated antibody (1:10,000; Invitrogen, Eugene, OR, USA, Cat#:A32735 and Cat#A32730) for 60 min. Target bands were captured and quantified using a fluorescence scanner (Odyssey Infrared Imaging System, LI-COR Biotechnology, Lincoln, NE, USA).

### Immunofluorescence

Immunofluorescence was performed to quantify the expressions of BDNF and VGF in the hippocampus and PFC, respectively. Mice (5 per group) were anesthetized with pentobarbital and then transcardially perfused with cold 0.9% saline, and their brains were removed and fixed in phosphate-buffered 4% paraformaldehyde. Mouse brain coronal sections of the hippocampus and PFC (30-µm thick) were collected using a cryostat (Leica, Wetzlar, Germany) and were first permeabilized with 2% Triton X-100 in PBS for 30 min, then incubated with PBS containing 5% donkey serum for 1 h at room temperature, followed by incubation in diluted primary antibodies used for immunostaining including VGF (1:400, Abcam, Cambridge, MA, USA, Cat#ab115609) and BDNF (1:800, Abcam, Cambridge, MA, USA, Cat#ab108319) overnight at 4 °C. Twenty-four hours later, the primary antibodies were removed and washed in PBS and then incubated with secondary antibodies (donkey anti-rabbit conjugated with Alexa Fluor 488 [1:1000, Abcam, Cambridge, MA, USA, Cat#ab150073] and donkey anti-rabbit conjugated with Alexa Fluor 594 [1:1000, Abcam, Cambridge, MA, USA, Cat#ab150076]) in blocking buffer for 1 h at room temperature. Brain sections were then stained with 4,6-diamidino-2-phenylindole (DAPI) for 15 min, mounted onto slides, and coverslipped with ProLong Gold Antifade Mountant (Invitrogen). The images were analyzed using a confocal laser scanning microscope (LSM710, Zeiss, Germany).

### Statistical analyses

All data statistical analyses were performed using GraphPad Prism (Version 5.0, Prism software for PC, GraphPad) and are presented as means ± standard errors of the mean (SEM). The significance was assessed by one-way ANOVA followed by Bonferroni multiple comparison. *P* < 0.05 was considered statistically significant.

## Results

### The antidepressant-like effects of repeated treatment with rapastinel in the FST of mice

To confirm the long-lasting effects of the repeated treatment with rapastinel, we further compared it with the antidepressant-like actions of a single dose in the FST of mice. Figure 1A, B show the experimental timeline schematic. Our data found that repeated treatment with rapastinel significantly decreased the immobility time at 1 (*P* < 0.01), 7 (*P* < 0.01), and 14 days (*P* < 0.01) after withdrawal of rapastinel (Fig. 1C). However, single treatment with rapastinel produced significant inhibition in the immobility time only at 1 day after withdrawal of rapastinel (Fig. 1D). Our data indicated that repeated treatment with rapastinel induced antidepressant-like effects in the FST of mice. However, single treatment with rapastinel induced antidepressant-like effects that lasted less than 1 week.

### The antidepressant-like effects of repeated treatment with rapastinel dependent on the VGF/BDNF/TrkB/ERK pathway in mice

**Fig. 2:**
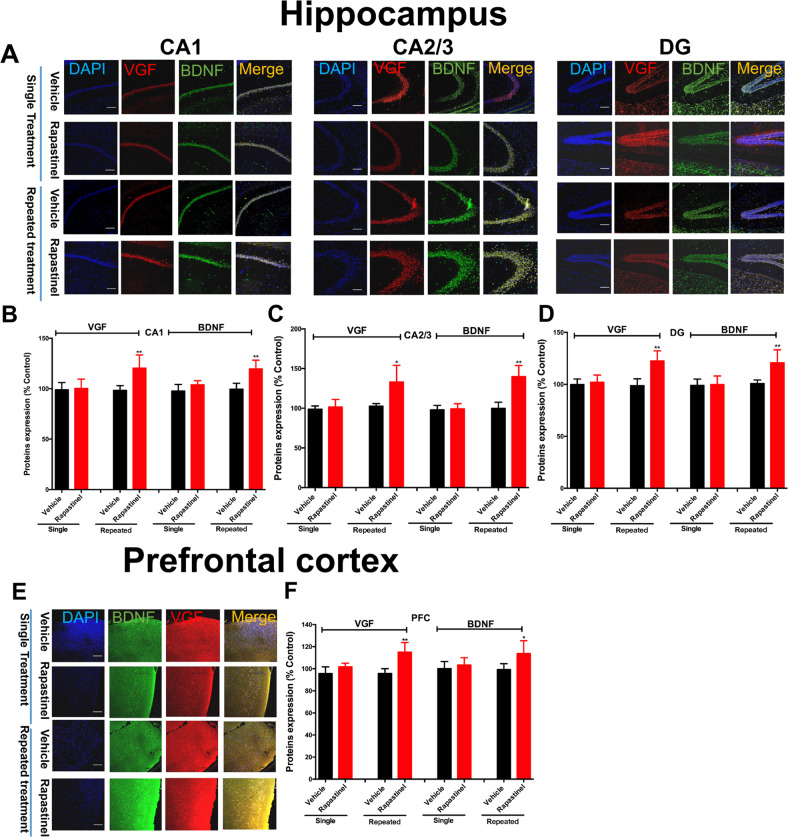
Repeated treatment with rapastinel upregulates the expressions of BDNF and VGF in the hippocampus and PFC of mice. Immunofluorescent images of BDNF and VGF in the CA1, CA2/3, and DG subregions of the hippocampus (**A**) and PFC (**E**) in mice. Quantification of BDNF and VGF expression in the CA1, CA2/3, and DG subregions of the hippocampus (**B**, **C**, and **D**) and PFC (**F**) in mice. Repeated treatment with rapastinel produced long-lasting upregulation of the expression of BDNF (green) with VGF (red) in the hippocampal and PFC neurons in mice. Scale bar = 200 μm. Data are expressed as means ± S.E.M; *n* = 5 per group. *P < 0.05, **P < 0.01 compared with vehicle group.

Fourteen days after single or repeated treatment with rapastinel, the western blot results indicated that only the 3-day repeated treatment with rapastinel produced a significant increase of pERK [hippocampus: *P* < 0.01; PFC: *P* < 0.01; Fig. 1E, F], pmTOR [hippocampus: *P* < 0.01; PFC: *P* < 0.05; Fig. 1E, G], and pTrkB [hippocampus: *P* < 0.05; PFC: *P* < 0.05; Fig. 1E, H] in the hippocampus and PFC. In addition, the immunohistochemical results revealed long-lasting upregulation of BDNF and VGF in the CA1 (BDNF, *P* < 0.01, Fig. 2A, B; VGF, *P* < 0.01, Fig. 2A, B), CA2/3 (BDNF, *P* < 0.01, Fig. 2A, C; VGF, *P* < 0.05, Fig. 2A, C), and dentate gyrus (DG) (BDNF, P < 0.01, Fig. 2A, D; VGF, *P* < 0.01, Fig. 2A, D) subregions of the hippocampus and PFC (BDNF, *P* < 0.05, Fig. 2E, F; VGF, *P* < 0.01, Fig. 2E, F) by repeated rapastinel treatment in mice. However, single treatment did not show significant effects on BDNF and VGF in the hippocampus and PFC, respectively.

### Repeated rapastinel treatment produced rapid and antidepressant-like effects in susceptible mice after CUS

**Fig. 3:**
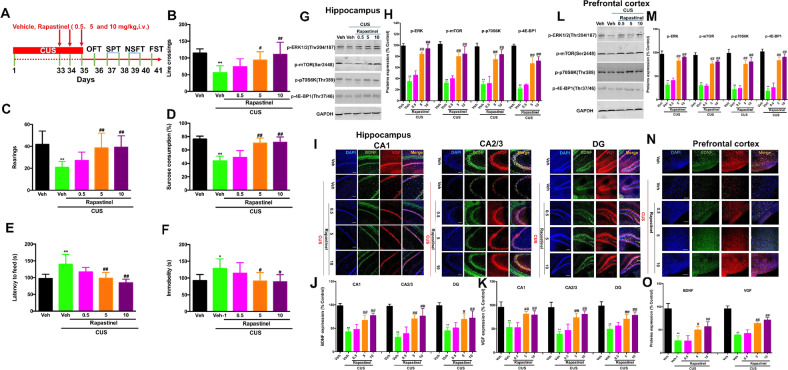
Repeated treatment with rapastinel produces rapid antidepressant effects in a CUS paradigm and significantly reversed the downregulation of VGF, BDNF, and ERK/mTOR signaling in mice. **A** Schematic demonstrating the timeline for CUS exposure, drug treatment, and behavioral tasks. **B**, **C** Reversed effects of rapastinel on CUS-induced decrease of locomotor activity in the OFT, as assessed by line crossings (**B**) and rearings (**C**). **D** Rapastinel treatment rapidly reverses anhedonia as measured by a reduction in 1% sucrose preference after CUS. **E** Rapastinel administration rapidly reverses the changes in latency to feed in the NSFT. **F** Rapastinel significantly prevented CUS-induced increase in immobility time in FST. **G**, **L** Representative western blot images; **H**, **M** Quantitative analysis of blots pERK, ERK, p-mTOR, p-p70S6K.p-4EBP1. Immunofluorescent images of BDNF and VGF in the CA1, CA2/3, and DG regions in the hippocampus (**I**) and PFC (**N**) of mice. Rapastinel reverses the CUS-induced downregulation of expression of BDNF (green) with VGF (red) in hippocampus (**J** and **K**) and PFC (**O**) neurons in mice. Scale bar = 200 μm. Quantification of BDNF and VGF expression in the hippocampus and PFC of mice. Data are expressed as means ± S.E.M; *n* = 9 for behavioral tasks, *n* = 3/mice for western blot and *n* = 5/mice for immunofluorescence; *P < 0.05, **P < 0.01 compared with the vehicle + non-stressed group; #*P* < 0.05, ##*P* < 0.01 compared with the vehicle + CUS group.

We examined whether repeated treatment with rapastinel demonstrated rapid and long-lasting effects in the CUS model. The 35-day CUS procedure was used to induce the depression mouse model (the timeline has been shown in Fig. 3A). The mice received three consecutive intraperitoneal (i.p.) injections of rapastinel (0.5, 5, and 10 mg/kg, i.v.), or its vehicle from days 34 to 35, beginning from 1 h after the stressor session. Behavioral indicators were then assessed, including the locomotor activities in the OFT (day 36), sucrose intake in the SPT (day 37), latency to feed in the NSFT (day 39), and immobility duration in the FST (day 41).

Our data showed that CUS paradigm substantially decreased the line crossings (*F* (4, 40) = 9.088, *P* < 0.01, Fig. 3B) and rearings (*F* (4, 40) = 7.005, *P* < 0.01, Fig. 3C), and this alteration was significantly reversed by rapastinel (5 and 10 mg/kg) treatment. Additionally, the CUS paradigm substantially decreased sucrose preference (*F* (4, 40) = 34.04, *P* < 0.01, Fig. 3D), which was reversed by rapastinel (5 and 10 mg/kg) treatment. A 35-day CUS paradigm induced a significant increase in the latency to feed in the NSFT than in non-stressed mice (*F* (4, 40) = 12.76, *P* < 0.01, Fig. 3E). However, rapastinel (5 and 10 mg/kg) treatment significantly reversed these effects. Furthermore, the immobility time was significantly increased by CUS in the FST compared with that in non-stressed mice (*F* (4, 40) = 4.362, *P* < 0.05, Fig. 3F). However, rapastinel (5 and 10 mg/kg) treatment significantly reversed these effects.

### Repeated rapastinel treatment significantly reversed the CUS-induced downregulation of ERK/mTOR/BDNF/VGF signaling in the hippocampus and PFC of mice

The effects of rapastinel on the expressions of p-ERK1/2, ERK1/2, p-mTOR, p-p70S6k, p-4E-BP1, BDNF, and VGF in the CUS-exposed mice were observed in the hippocampus (Fig. 3G, H) and PFC (Fig. 3L, M), respectively. Thirty-five days of CUS exposure induced a significant reduction in the levels of p-ERK1/2 (hippocampus, *F* (4, 10) = 62.32, *P* < 0.01, Fig. 3H; PFC: *F* (4,10) = 119.5, *P* < 0.01, Fig. 3M), p-mTOR (hippocampus, *F* (4, 10) = 48.25, *P* < 0.01, Fig. 3H; PFC: *F* (4,10) = 147.9, *P* < 0.01, Fig. 3M), p-p70S6k (hippocampus, *F* (4, 10) = 38.30, *P* < 0.01, Fig. 3H; PFC: *F* (4, 10) = 60.29, *P* < 0.01, Fig. 3M), and p-4E-BP1 (hippocampus, *F* (4, 10) = 79.96, *P* < 0.01, Fig. 3H; PFC: *F* (4, 10) = 86.71, *P* < 0.01, Fig. 3M), and these reductions were significantly reversed by repeated rapastinel treatment. However, the total ERK1/2 did not vary significantly among any groups (not shown). Additionally, we examined the expression of BDNF and VGF in the subregions of the hippocampus (Fig. 3I–K) and PFC (Fig. 3N, O) and observed that the decreases in BDNF (As shown in 3J, CA1, *F* (4,20) = 40.42, *P* < 0.01; CA2/3, *F* (4,20) = 31.75, *P* < 0.01; DG, *F* (4,20) = 18.39, *P* < 0.01; As shown in 3O, PFC: *F* (4,20) = 38.42, *P* < 0.01) and VGF (As shown in 3K, CA1, *F* (4,20) = 19.92, *P* < 0.01; CA2/3, *F* (4,20) = 29.44, *P* < 0.01; DG, F (4,20) = 27.72, *P* < 0.01; As shown in 3O, PFC: *F* (4,20) = 91.80, *P* < 0.01) were significantly reversed by repeated rapastinel treatment in mice.

### Inhibition of ERK signaling significantly abolished the antidepressant-like actions of repeated treatment with rapastinel in mice

**Fig. 4:**
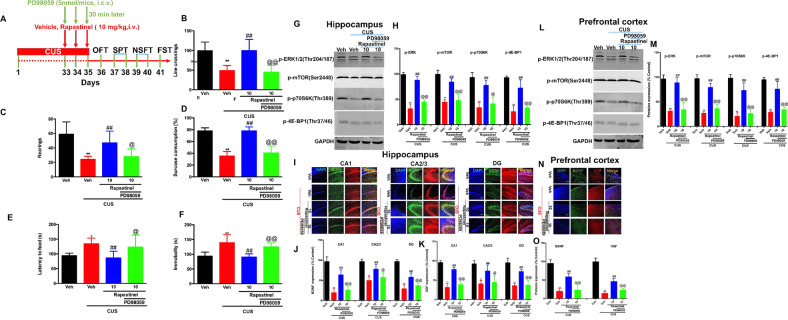
Inhibition of ERK signaling significantly abolishes the antidepressant-like actions and upregulation of VGF, BDNF, and ERK/mTOR signaling produced by repeated treatment with rapastinel in mice. **A** Schematic demonstrating the timeline for cannula implanted, CUS exposure, drug treatment, and behavioral tasks. **B**, **C** Pretreatment with PD98059 significantly alleviated effects of rapastinel on CUS-induced decrease of locomotor activity in the OFT, as assessed by line crossings (**B**) and rearings (**C**). **D** Inhibition of ERK signaling blocks the rapastinel treatment rapidly reversed anhedonia as measured by a reduction in 1% sucrose preference after CUS. **E** Rapastinel administration rapidly reversed the changes in latency to feed in the NSFT. **F** Rapastinel significantly prevented CUS-induced increase in immobility time in FST. **G** and **L** Representative western blot images; **H** and **M** Quantitative analysis of blots (p-ERK, ERK, p-mTOR, p-p70S6K, and p-4E-BP1). **C** Immunofluorescent images of BDNF and VGF in the CA1, CA2/3, and DG regions in the hippocampus (**I**) and PFC (**N**) of mice. Rapastinel reversed the CUS-induced downregulation of expression of BDNF (green) with VGF (red) in hippocampus (**J** and **K**) and PFC (**O**) neurons in mice. PD98059 can abolish the antidepressant effect of rapastinel in the expression of BDNF (green) and VGF (red) in hippocampus and PFC neurons in mice. Scale bar = 200 μm. Data are expressed as means ± S.E.M; *n* = 9 for behavioral tasks, *n* = 3/mice for western blot and *n* = 5/mice for immunofluorescence; **P < 0.01 compared with the vehicle + non-stressed group; ##*P* < 0.01 compared with the vehicle + CUS group; @*P* < 0.05, @@*P* < 0.01 compared with the rapastinel + CUS group.

We explored whether ERK-mediated signaling was required for repeated treatment with rapastinel-produced rapid antidepressant-like effects and the expression of BDNF and VGF in mice (the timeline has been shown in Fig. 4A). We found that rapastinel significantly reversed the downregulation of the line crossings [*F* (3, 32) = 19.07, *P* < 0.01, Fig. 4B] and rearings [*F* (3, 32) = 14.02, *P* < 0.01, Fig. 4C] induced by CUS exposure. However, pretreatment with PD98059 significantly restored the effects of rapastinel on line crossings (*P* < 0.01) and rearings (*P* < 0.05). Additionally, for sucrose preference in the SPT, the increase in sucrose consumption by rapastinel was completely blocked by pretreatment with the antagonist PD98059 (*F* (3, 32) = 73.17, *P* < 0.01, Fig. 4D). Further, for the feeding latency in the NSFT, pretreatment with PD98059 significantly blocked the decrease in latency to feed produced by rapastinel (*F* (3, 32) = 7.151, *P* < 0.05, Fig. 4E). Additionally, the home-cage feed consumption for 6 min was similar in each group (data not shown). Finally, we investigated whether PD98059 blocked rapastinel-induced antidepressant-like activity using FST. Likewise, PD98059 pre-administration significantly eliminated immobility time reduction due to repeated rapastinel treatment (*F* (3, 32) = 17.87, *P* < 0.01, Fig. 4F).

### Inhibition of ERK significantly abolished the upregulation of repeated treatment with rapastinel on the p-ERK1/2, p-mTOR, p-p70S6k, p-4EBP1, BDNF, and VGF in the hippocampus and PFC of mice

Western blot results indicate that rapastinel (10 mg/kg) significantly reversed the decrease in p-ERK1/2 (hippocampus: *F* (3, 8) = 69.28, *P* < 0.01, Fig. 4G, H; PFC: *F* (3,8) = 36.07, *P* < 0.01, Fig. 4L, M), p-mTOR (hippocampus, *F* (3, 8) = 31.93, *P* < 0.01, Fig. 4G, H; PFC: *F* (3,8) = 63.17, *P* < 0.01, Fig. 4L, M), p-p70S6k (hippocampus, *F* (3, 8) = 20.25, *P* < 0.01, Fig. 4G, H; PFC: *F* (3,8) = 40.35, *P* < 0.01, Fig. 4L, M), and p-4EBP1 (hippocampus: *F* (3, 8) = 29.22, *P* < 0.01, Fig. 4G, H; PFC: *F* (3,8) = 41.44, *P* < 0.01, Fig. 4L, M) induced by CUS in mice. However, PD98059 pretreatment significantly blocked the effects of rapastinel (all *P* < 0.01). No significant alteration was noted in total ERK1/2 levels in any group (data was not shown). Additionally, the immunohistochemical results revealed that the downregulation of BDNF and VGF in the CA1 (BDNF, *F* (3,16) = 55.98, *P* < 0.01, Fig. 4I and Fig. 4J; VGF, *F* (3,16) = 88.99, *P* < 0.01, Fig. 4I, K), CA2/3 (BDNF, *F* (3,16) = 24.12, *P* < 0.01, Fig. 4I, J; VGF, *F* (3,16) = 16.33, *P* < 0.01, Fig. 4I, K), and DG (BDNF, *F* (3,16) = 78.43, *P* < 0.01, Fig. 4I, J; VGF, *F* (3,16) = 43.77, *P* < 0.01, Fig. 4I, K) subregions of the hippocampus and PFC (BDNF, *F* (3,16) = 59.42, *P* < 0.01, Fig. 4N, O; VGF, *F* (3,16) = 137.3, *P* < 0.01, Fig. 4N, O) by CUS were reversed by repeated rapastinel treatment in mice. However, these changes were significantly blocked by pretreatment with PD98059 in mice.

## Discussion

Current therapeutic options for MDD are associated with a lag in onset that can prolong distress and increase the risk of suicide in patients, and their antidepressant efficacy is often limited. More and more clinical and preclinical studies strongly suggest that the exploration and discovery of new rapid antidepressants is a hotspot of current research. Recently, promising clinical findings have been reported for several compounds, including esketamine and another allosteric modulator of NMDAR, rapastinel [14, 40–42]. Although four of the five esketamine phase III clinical trials were negative, the Food and Drug Administration (FDA) has provided approval based on one positive trial and promising data in another trial. Similar to ketamine, rapastinel has produced rapid antidepressant actions in phase II clinical trials [14] and in preclinical rodent models [13, 16–18]. Importantly, rapastinel did not induce memory deficits and psychotomimetic dysfunctions. However, disappointingly, negative findings were observed in four phase III trials (ClinicalTrials.gov Identifiers NCT02932943, NCT02943564, NCT02943577, NCT02951988). In 2019, Allergan plc (NYSE: AGN) announced topline results from three pivotal studies of rapastinel as an adjunctive treatment of MDD. Despite the failure of the clinical trials, exploring the cause of the failure is critical. Therefore, further preclinical studies are required before finally determining the efficacy of the agent [43]. The previous reports demonstrate that rapastinel is a weak modulator that enhances NMDAR activity at antidepressant-like concentrations and inhibits NMDAR activity at higher concentrations [15], indicating that rapastinel as an allosteric modulator of NMDAR. The allosteric modulation of NMDAR by rapastinel [15] may represents a novel pharmacological approach to promote well-tolerated, rapid, and sustained improvements in depression. According to pharmacological theory, the action of allosteric modulator is related to the dose of the drug and the pile of the receptors. In three acute clinical studies (ClinicalTrials.gov Identifiers NCT02932943, NCT02943564, NCT02943577) and an interim analysis of the rapastinel relapse prevention study (NCT02951988), the rapastinel treatment arms did not differentiate from placebo on the primary and key secondary endpoints.These studies completed the acute clinical trails of the efficacy, safety, and tolerability of the dose of rapastinel 450 mg IV once weekly (A 3-week double-blind treatment period) versus placebo as an adjunctive to antidepressant therapy (ADT) in patients with MDD (NCT02932943, NCT02943564, NCT02943577) who have a partial response to ADT. In addition, the long-term studies [8 to 16 weeks open label treatment period (OLTP), followed by a randomized double-blind treatment period (DBTP) of up to 104 weeks] about the efficacy, safety and tolerability of rapastinel 450 mg IV once weekly or once every 2 weeks versus placebo as an adjunctive treatment to ongoing ADT in the prevention of relapse in participants with MDD. The used 450 mg unit dose in these clinical trails is expected to be appropriate for most patients as this represents a dose of 4.5 mg/kg in a 100 kg patient. Based on the practice guide [44] for dose conversion between animals and human,the human dose (mg/kg) to mouse dose (mg/kg) should be multiplied by 12.3 (4.5 mg/kg*12.3 = 55.35 mg/kg), which are not remotely similar to the current doses used in mouse. We used doses in the mouse have been proved effective in the previus preclinical studies. The doses from animals to humans needs consideration of body surface area, pharmacokinetics, and physiological time, which may explain on of the reasons for the inconsistent results. The actions of allosteric modulator of rapastinel is related to the doses of the rapastinel, the duration of treatment and the pile of the receptors, which may also explain the potential inconsistent results. Further studies should be conduced to explore the novel of molecular mechanisms in different doses and treatment duration that underlie the antidepressant actions of rapastinel in the future.

In the present study, we first compared the potential mechanisms between single and repeated infusions of rapastinel in mice. To determine the duration of the effect of repeated or single treatment with rapastinel, we exposed mice to 3-day or a single-dose treatment and evaluated the immobility time at 1, 7, and 14 days after drug treatment cessation. Furthermore, our data further indicated that 3-day repeated rapastinel treatment produced antidepressant-like actions (14 days), accompanied by potentiation of ERK/mTOR/VGF/BDNF/TrkB signaling. However, a single-dose treatment with rapastinel induced antidepressant-like actions only within 1 week. Comprehensive analysis of our findings, in the short-term, revealed that repeated rapastinel therapy may be a better dosage regimen to maintain and to extend its antidepressant effects dependent on ERK/mTOR/VGF/BDNF/TrkB signaling. Our results shown that continuous 3-day rapastinel produced significantly antidepressant-like actions. In consistent with the recently work, which found that repeated administration of rapastinel produces exceptionally prolonged rescue of memory deficits in phencyclidine-treated mice [45]. Given that our current work shown that the single treatment with rapastinel induced antidepressant-like effects that lasted only less than 1 week, further studies will be required to determine whether a weekly dosing protocol also as the optimal choice. Moreover, we observed that a single rapastinel treatment reversed the chronic social defeat stress-induced depression-like behaviors in mice (Supplementary Fig. S1). Additionally, the rapid antidepressant actions of rapastinel require VGF/TrkB activation dependent on α-amino-3-hydroxy-5-methyl-4-isoxazolepropionic acid receptor (AMPAR) activation (NBQX-sensitive) (Supplementary Fig. S2), consistent with the findings of our previous report, which revealed that AMPAR-mediated VGF in the hippocampus plays a key role in the rapid-acting antidepressant-like actions of rapastinel in mice [18]. Our findings indicated that AMPAR activation and release of VGF and BDNF are required for the rapid antidepressant effects of rapastinel in mice. Previous reports have shown the drug to readily cross the blood–brain barrier, showing a brain uptake index of 80% [46, 47]. In addition, the effects of rapastinel were sustained for much longer than its half-life in rats [19] and dogs (plasma half-life of rapastinel is no more than 10 min). Our findings may suggest that short-term repeated treatment with rapastinel may extend the antidepressant effect of rapastinel dependent on the ERK/mTOR/VGF/BDNF/TrkB signaling.

To confirm the dose-dependent effect of rapastinel on the antidepressant actions and enhancing of the ERK/mTOR/VGF/BDNF/TrkB signaling, we aimed to investigate the antidepressant effect of rapastinel in the CUS model after 3-day repeated administrations. We examined the antidepressant-like effects of rapastinel, used as a reference compound at three doses (0.5, 5, and 10 mg/kg), based on a previous report for testing rapid antidepressant effects in mice. Consistent with our previous study [18], our present study provided further evidence that rapastinel ameliorates depression-like behaviors in CUS-induced mice in the SPT, NSFT, and FST. Our current work further confirmed that rapastinel rapid antidepressant-like effect in a dose-dependent manner.

As reported previously, the exceptional profiles of the antidepressant-like activity of rapid antidepressants were related to the immediate activation of molecular processes that lead to enhanced neuroplasticity, mainly in the PFC and hippocampus [29, 34, 48, 49], which are functionally impaired in MDD. Multiple mechanisms have been proposed to explain rapastinel’s rapid antidepressant actions. More importantly, we demonstrated that repeated treatment with rapastinel significantly reversed the CUS-induced downregulation of p-ERK1/2, p-p70S6K, and p-4EBP1 in the PFC and hippocampus of mice. In this study, we further observed a marked reduction in BDNF and VGF protein levels in the PFC, CA1, CA2/3, and DG regions of the hippocampus of depressed mice after CUS, consistent with recent results from previous reports, indicating that ERK/mTOR/VGF/BDNF signaling may be involved in the antidepressant-like effects of rapastinel.

Increasing evidence supports a pivotal role of the ERK subclass of mitogen-activated protein kinase (MAPK), especially ERK1/2, which has been most thoroughly investigated and characterized in the PFC and hippocampus of depressed animals and has played a pivotal role in various neuropsychiatric disorders, including MDD [50–52]. Here, we observed that the 3-day repeated treatment with rapastinel significantly increased phosphorylated ERK and mTOR levels, as well as downstream proteins, leading to increased synthesis of synaptic proteins (i.e., p70S6K and 4EBP1 kinase), which are downstream of mTOR signaling. Our results confirmed that the antidepressant-like effects of rapastinel in stress and depression may correlate with ERK/mTOR signaling. Previous studies indicate that the activation of the ERK signaling pathway is mediated by the TrkB receptor [53], which is regulated by BDNF, VGF, and VGF-derived C-terminal neuropeptides. It has been proposed that local translation and activity-dependent release of BDNF [54, 55] and VGF and VGF-derived C-terminal neuropeptides are involved in the antidepressant-like actions of antidepressants (i.e., ketamine, rapastinel, and scopolamine) [18, 31, 33]. Our present results demonstrated that CUS downregulated the expression of VGF and BDNF in the PFC and hippocampus and was significantly reversed by rapastinel. Multiple lines of evidence suggest that BDNF and its receptor TrkB play a crucial role in depression and in the mechanisms of antidepressants [29, 30]. Interestingly, VGF and its derived C-terminal neuropeptides also produce antidepressant-like effects dependent on the receptor of TrkB [30]. The involvement of TrkB in regulating antidepressant-like effects via ERK is highly correlated with synaptic plasticity [56, 57]. Our results demonstrate that repeated rapastinel injection could alleviate depression-like behaviors similar with ketamine binding to NMDAR, which further activated the VGF/BDNF/TrkB/ERK pathway, thereby regulating protein translation (e.g., VGF and BDNF). Recently reviews [11, 58] indicated that the antidepressant mechanisms of ketamine may including synaptic or GluN2B-selective extra-synaptic NMDAR inhibition, inhibition of NMDAR localized on GABAergic interneurons, inhibition of NMDAR-dependent burst firing of lateral habenula neurons, and the role of AMPAR activation. In addition, the finding of the antidepressant actions of ketamine required activity of a distinct metabolite and is independent of NMDAR inhibition, indicate a novel mechanism underlying ketamine’s unique antidepressant properties [59]. However, the early and sustained activation of AMPAR and release of BDNF may as the one partly mechanisms of ketamine and it’s metabolites in the rapid antidepressant actions [11, 59]. However, rapastinel as an allosteric modulator of NMDAR is a novel glutamatergic compound. In contrast to ketamine, recently study [15] revealed that rapastinel at therapeutic concentrations directly enhances NMDAR through a novel site independent of the glycine co-agonist site and produces distinct behavioral, sleep, and EEG profifiles compared with ketamine [60]. Herein, our results further confirmed that the BDNF and VGF release may involve in the antidepressant-like effects of rapastinel, which has been shown to directly enhance NMDAR activity via a novel domain independent of the glycine co-agonist site [15]. Given that rapastinel and ketamine with a distinct initial trigger but lead to convergent antidepressant effects via activation of the AMPAR, further BDNF and VGF release, and TrkB-mTOR pathway, suggesting that repastinel is partially similar to ketamine, and the potential downstream regulation need to be further explore.

**Fig. 5:**
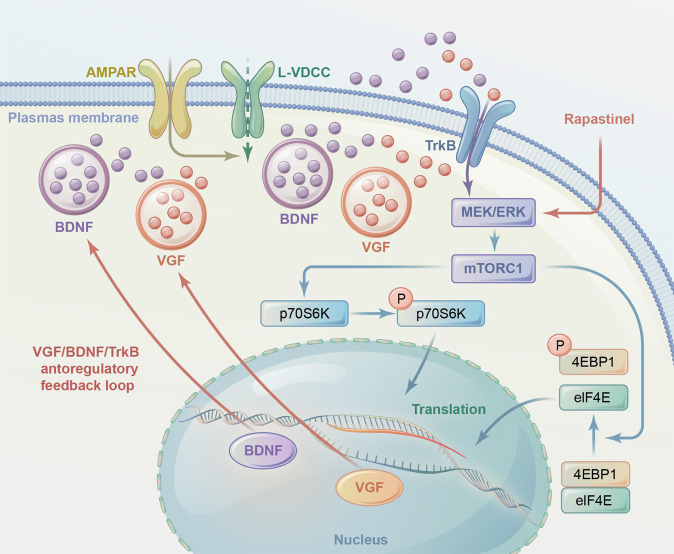
The working model of the antidepressant-like actions of single or repeated treatment with rapastinel in mice. Our results showed ERK-mediated mTOR signaling may play critical in the antidepressant-like effects of repeated treatment with rapastinel in mice. Our data further indicated that these effects may be mediated partly through the ability of rapastinel to enhance VGF and BDNF expression and then the VGF/BDNF/TrkB autoregulatory feedback loop by increasing the ERK/mTOR pathway. The VGF/BDNF/TrkB autoregulatory feedback loop may supply the VGF and BDNF to facilitate the AMPAR-mediated fast releases in the rapid-acting antidepressant actions of single or repeated treatment with rapastinel.

In line with our expectations, the effects of rapastinel were significantly blocked by intracerebroventricular injection of the MEK/ERK inhibitor PD98059. The upregulation of phosphorylation of mTOR, ERK1/2, p70S6K, and 4EBP1 after repeated treatment with rapastinel in the hippocampus and PFC was abolished by PD98059. Additionally, the increase in VGF and BDNF levels by repeated rapastinel treatment was blocked by PD98059. The rapid and long-lasting antidepressant effects of rapastinel demonstrated that ERK/mTOR signaling cascades led to a significantly increase in protein translation and protein synthesis (i.e., VGF and BDNF) and strengthened VGF/BDNF/TrkB signaling in a self-reinforcing autoregulatory loop that could be required for one potential mechanism underlying the efficacy of rapid-acting antidepressants [30]. Importantly, activated mTOR, p70S6K, and 4EBP1 are specific only for rapid antidepressants, not typical antidepressants (such as selective serotonin reuptake inhibitors or tricyclic antidepressants) [61–63]. Activation of mTOR contributes to the phosphorylated activation of two major downstream substrates: p70S6K and eukaryotic translation initiation factor 4EBP1, leading to the induction of VGF and BDNF translation and stability of the antidepressant-like effects of rapastinel (the working model was shown in Fig. 5). Similar with the ketamine, which one of the glutamatergic antagonists, is an uncompetitive NMDARs open-channel blocker, rapidly relieving depressive-like behaviors and exerting long-lasting antidepressant actions. Rapastinel as an allosteric modulator of NMDAR is a novel glutamatergic compound, which is partially similar to ketamine, and the potential downstream regulation need to be further explored. Our results directly indicated that ERK/mTOR-mediated VGF and BDNF were convergent downstream targets that underlie the behavioral actions of rapastinel, indicating that the activation of the VGF/BDNF/TrkB autoregulatory feedback loop led to increased transcription, synthesis, and/or secretion of VGF and BDNF and increased antidepressant behavioral effects of repeated rapastinel treatment. A growing body of evidence indicate that mTOR as the key “Star molecule” involve in the rapid-acting antidepressant actions and synaptogenesis [64–67]. In recent years, whether mTOR is involved in rapid antidepressant effect has also been controversial. Interestingly, recently clinical trail conducted the first in-human study to examine the role of mTORC1 on ketamine’s antidepressant effects, showing that rapamycin pretreatment did not alter the antidepressant effects of ketamine at the 24 h timepoint. Unexpectedly, over the subsequent 2-weeks, there was a rapamycin-induced prolonged durability of ketamine’s clinical benefit [68]. Given that rapastinel and ketamine with a distinct initial trigger on NMDAR but lead to convergent antidepressant effects via activation of mTOR pathway in preclinical study, the involvement of mTOR in the rapid-acting antidepressant effects of rapastinel need to be further explored in preclinical and clinical trails.

A growing body of evidence demonstrates that NMDAR-mediated synaptic plasticity and metaplasticity play important role in the pathophysiology and for antidepressant activity outlasting drug presence [22, 69, 70]. Our previous report [27] have demonstrated that the NMDAR subunit NR2B involve in the anti-schizophrenia-like phenotype by rapastinel in mice. In addition, the directly enhance NMDAR activity via a novel domain independent of the glycine co-agonist site by rapastinel was reported recently [15]. Importantly, previous data demonstrate that rapastinel produces its long-lasting antidepressant effects via triggering NR2B-dependent processes that lead to increased sensitivity to LTP that persist for up to 2 weeks, indicating that the long-lasting antidepressant effects of rapastinel are associated with a metaplasticity process in the medial prefrontal cortex and hippocampus [69]. In our current work, we demonstrated that the antidepressant actions of rapastinel dependent on release of BDNF and VGF, which they play critial role in the neuroplasticity and metaplasticity [71–73]. Our findings are consistent with previous studies demonstrating that structural plasticity of spine synapse formation requires BDNF release and activation of TrkB ignaling [74, 75]. It will be important in future studies to determine if the antidepressant-like actions of rapastinel also require activity-dependent ERK/mTOR/VGF/BDNF signaling-medicated synaptic plasticity and metaplasticity in the hippocampus and prefrontal cortex of mice.

In conclusion, our results indicated that repeated rapastinel treatment exerts significantly antidepressant-like effects in mice and the effects may be mediated partly through the ability of rapastinel to enhance VGF and BDNF expression and then the VGF/BDNF/TrkB autoregulatory feedback loop by increasing the ERK/mTOR pathway (The working model is shown in Fig. 5).

## Supplementary information

Supplementary data
