# Supplementary material for: ERK/mTOR signaling may underlying the antidepressant actions of rapastinel in mice
Source: Transl Psychiatry. 2022 Dec 22;12:522. doi: 10.1038/s41398-022-02290-5 (PMC9780240; doi:10.1038/s41398-022-02290-5)

**Supplementary Materials for**

**ERK/mTOR signaling may underlying the antidepressant actions of rapastinel in mice**

**This file includes:**

Materials and Methods

Table S1,Figure S1 to S2

Original gels of western blotting

**Materials and Methods**

**Animals**

Adult male C57BL/6J mice (weight: 20-25g at the first day of experiment) were reared in the animal facility of Ningbo University School of Medicine, China. All animals were maintained at 22°C ± 2°C and 60% ± 5% relative humidity under a 12-h-light/-dark cycle (lights on at 7:00 am) with ad libitum access to food and water when the stressors were not applied. All stressors were applied to animals outside their housing areas in a separate procedure room. All procedures involving animals were conducted in accordance with the National Institute of Health Guidelines for the Care and Use of Laboratory Animals (NIH Publications No. 80-23, revised 1996) and the European Community Council Directive for the Care and Use of Laboratory Animals of September 22, 2010 (2010/63/EU). All experiments were approved by the Institutional Animal Care and Use Committee of Ningbo University, School of Medicine.

**Table S1 Chronic unpredictable stress paradigm in mice**

| **Stressors** | **Duration** | **Day** |
| --- | --- | --- |
| Food deprivation  Water deprivation  Physical restraint  Shock cage  Forced swimming at 40℃ | 24 h  24 h  3 h  1 h  5 min | Monday (Week 1)  Monday (Week 2)  Monday (Week 3)  Monday (Week 4)  Monday (Week 5) |
| Soiled cage (24 h)  Forced swimming at 10℃  Cage tilt (45℃)  Electric foot shock (5 min)  Overnight illumination | 24 h  5 min  24 h  5 min  24 h | Tuesday (Week 1)  Tuesday (Week 2)  Tuesday (Week 3)  Tuesday (Week 4)  Tuesday (Week 5) |
| Stroboscopic light and noise  Forced swimming at 10℃  Physical restraint  Overnight illumination  Food deprivation | 5 min  5 min  3 h  24 h  24 h | Wednesday (Week 1)  Wednesday (Week 2)  Wednesday (Week 3)  Wednesday (Week 4)  Wednesday (Week 5) |
| Water deprivation  Electric foot-shock  Overnight illumination  Soiled cage  Water deprivation | 24 h  5 min  24 h  24 h  24 h | Thursday (Week 1)  Thursday (Week 2)  Thursday (Week 3)  Thursday (Week 4)  Thursday (Week 5) |
| Cage tilt  Forced swimming at 40℃  Electric foot-shock  Stroboscopic light and noise  Forced swimming at 10℃ | 24 h  5 min  5 min  5 min  5 min | Friday (Week 1)  Friday (Week 2)  Friday (Week 3)  Friday (Week 4)  Friday (Week 5) |
| Electric foot-shock  Overnight illumination  Forced swimming at 40℃  Food deprivation  Water deprivation | 5 min  24 h  5 min  24 h  24 h | Saturday (Week 1)  Saturday (Week 2)  Saturday (Week 3)  Saturday (Week 4)  Saturday (Week 5) |
| Cage tilt (45℃)  Soiled cage  Forced swimming at 10℃  Overnight illumination  Stroboscopic light and noise | 24 h  24 h  5 min  24 h  5 min | Sunday (Week 1)  Sunday (Week 2)  Sunday (Week 3)  Sunday (Week 4)  Sunday (Week 5) |

**Drugs and stereotaxic intracranial injections**

The following drugs were used: rapastinel (10mg/kg, i.v.Tocris Bioscience). These solutions were dissolved in 0.9% saline, freshly prepared before administration.Mice were anesthetized in an induction box with 3.5% isoflurane and and subsequently maintained with 2.5% isoflurane through a nose cone and placed in a stereotaxic frame. The infusion cannula was implanted bilaterally into the prefrontal cortex (PFC) space according the fellowing bregma coordinated: AP：+1.78 mm，ML：±0.30mm， DV：-1.25 mm.The infusion cannula was implanted bilaterally for the intra-PFC microinjections, 2,3-Dihydroxy-6-nitro-7-sulfamoyl-benzo [f] quinoxaline-2,3-dione (NBQX) (Sigma-Aldrich, St. Louis, MO, USA) was dissolved in ACSF and microinjected at the dose of 10 nmol/1 μL. Animals were housed individually and allowed 7 days for recovery.

**Chronic social defeat stress（CSDS）**

Briefly, aggressive resident CD-1 mice selected on the basis of their attack latencies reliably shorter than 30 s on three consecutive screening tests. The experimental C57BL/6 J mouse was exposed to a different CD-1 aggressor mouse each day for 10 min for 10 days. After the 10 min physical contact, test mice were separated from the aggressor and were placed a plastic divider with holes, where they were exposed to sensory contact with CD-1 mice for the another 24 h. Control mice were housed in similar cages but with members of the same strain, which changed daily. 24 h after the last defeat, all mice were housed individually and then subjected to behavioral tasks.

**Open Field Test (OFT)**

The OFT is commonly used as the measurement of locomotor activity in rodents. We used a white square Plexiglas box (50 × 50 × 40 cm) and was divided by two cross black lines drawn on the floor. Mice were placed in the arena and allowed to explore for 5 min. The number of the line crossings and rearings were considered parameters of locomotor activity and recorded over the 5-min period by digital system.

**Sucrose preference test (SPT)**

SPT is a method to evaluate anhedonic-like behavior in animals based on sucrose taste preference. All were trained to adapt to the two bottles, one containing 1% sucrose solution and the other with fresh water for 24 h. The positions of the two bottles were randomly placed and were exchanged to prevent the formation of position preference. After adaptation, the mice were deprived of water for 24 h. The mice were housed in individual cages and had free access to two bottles containing sucrose solution and water. After 16 h, the volumes of the sucrose and water were measured and the sucrose preference rate was calculated as a percentage of the amount of sucrose intake/(water intake + sucrose intake) × 100%.

**Forced swimming test (FST)**

The FST was conducted in a sound-attenuated room. Briefly, mice were placed individually for 6 min into a clear plastic cylinder (diameter 10 cm, height 25 cm) containing 10 cm of fresh water, maintained at 23 ± 2 °C. The immobility time was recorded the last 4 min of the 6 min testing duration. The total time during which the mouse floated with no additional activity other than that necessary to keep the animal’s head above the water was considered the duration of immobility.

**Immunohistochemistry**

Immunohistochemistry was performed to quantify the densities of BDNF and VGF (the cells showing positive staining)in the PFC. Mice (n = 5 per group) were anesthetized under pentobarbital and then transcardially perfused with 250 mL cold saline solution followed by 250 mL of 4% PBS. Serial coronal sections of the hippocampus and PFC (30 µm thick) were collected on a cryostat (Leica, Wetzlar, Germany). The sections were first permeabilized with 2% TritonX-100 in PBS for 30 minutes, then incubated with PBS containing 5% donkey serum for another 1h at room temperature and followed by incubation in diluted anti-VGF (1:400, Invitrogen, USA, Cat#MA5-41690) and anti-pTrkB (1:800, Sigma-Aldrich, USA, Cat#ABN1381) antibodies overnight at 4℃. 24 hours later, the primary antibodies were removed and washed in the PBS and then incubated with fluorescent secondary antibodies, donkey anti rabbit conjugated onjugated with Alexa Fluor 488 (1:1000, Abcam, Cambridge, MA, USA,Cat#ab150073) and donkey anti-mouse conjugated with Alexa Fluor 594 (1:1000, Thermo Fisher Scientific, MA, USA, Cat#R37115) for 1 hour at room temperature. DNA (nuclei) was stained with 4′,6-diamidino-2-phenylindole (DAPI) for 15 min, mounted onto slides, and coverslipped with Pro Long Gold Antifade Mountant (Invitrogen). The images wereanalyzed using a confocal laser-scanning microscope (LSM710, Zeiss, Germany).

**Statistical Analysis**

All data statistical analyses were performed using GraphPad Prism (Version 5.0, Prism software for PC, GraphPad) and are presented as mean ± standard error of the mean (SEM). The significance was assessed by one-way ANOVA and fellowed by Bonferroni multiple comparison P< 0.05 were considered statistically significant.

**Supplementary Figures**

**
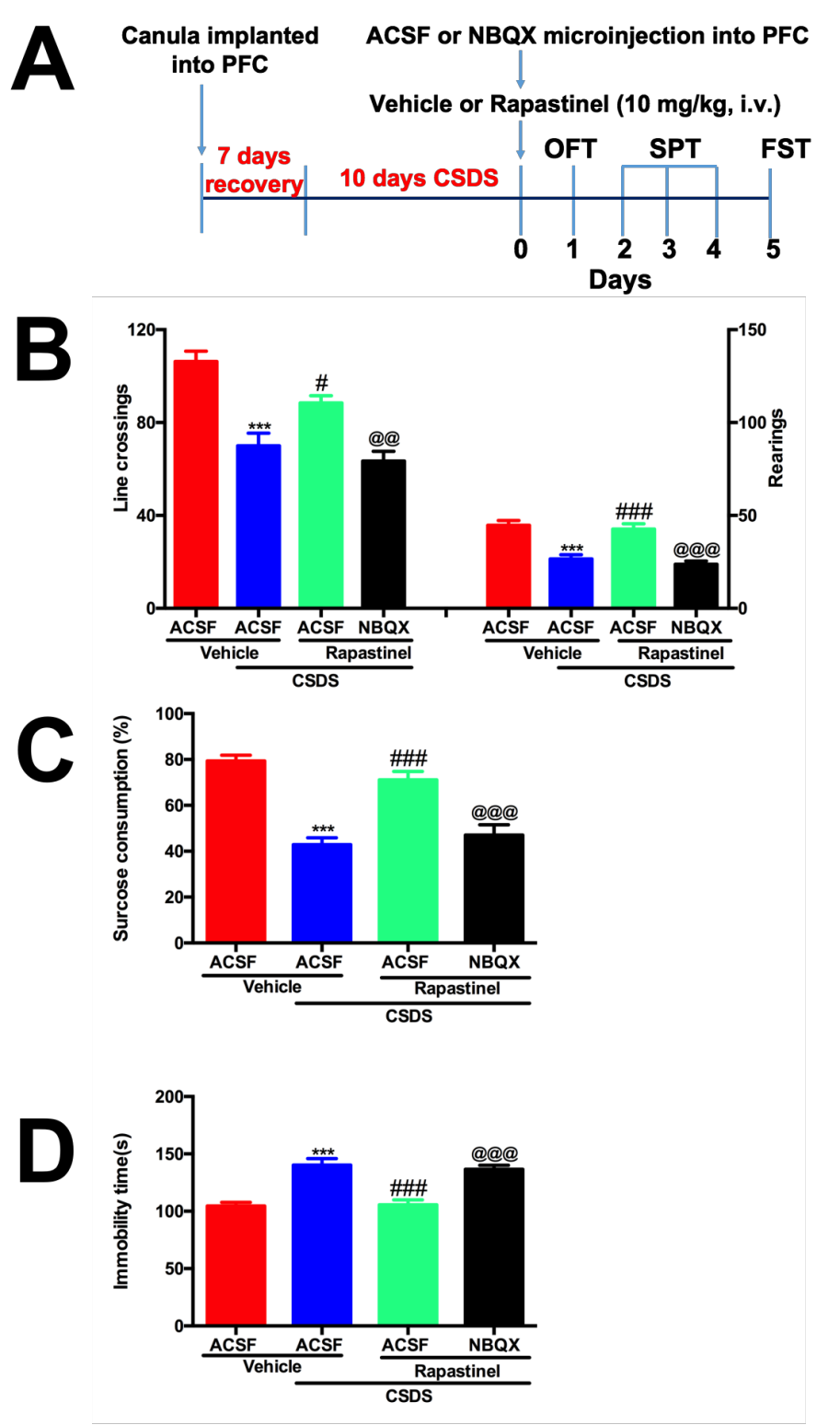
**

**Figure S1. Pretreatment with an AMPAR antagonist into PFC blocks rapid antidepressant-like actions of rapastinel in mice.**

Our previous report which have reveal that the AMPAR-mediated VGF in hippocampus plays key role in the rapid-acting antidepressant-like actions of rapastinel in mice. To further examine whether the AMPAR-mediated VGF release and activation of TrkB in the PFC involved in the rapid antidepressant-like effects by single treatment with rapastinel, we used the OFT, SPT and FST to detect locomotor activies, anhedonia and despair behaviors in mice respectively. (A) Schematic demonstrating the timeline for CSDS exposure, drug injection, and behavioral testing. Numbers in parentheses represent days after drug injection.The CSDS is a well-validated animal model that induces depressive behaviors in mice and single treatment with rapastinel significantly reversed the depressive-like effects in the OFT (B), SPT (C) and FST (D) within one week. However, injection the AMPAR antagonist NBQX abolished the rapid-acting antidepressant-like effects of rapastinel. n= 5 per group. ***P< 0.001 vs. Vehicle + ACSF group; #P < 0.05, ###P < 0.001 vs. CSDS + Vehicle + ACSF group; @@P < 0.01, @@@P < 0.001 vs. CSDS + Rapastinel + ACSF group*.* All data are mean±SEM.

**
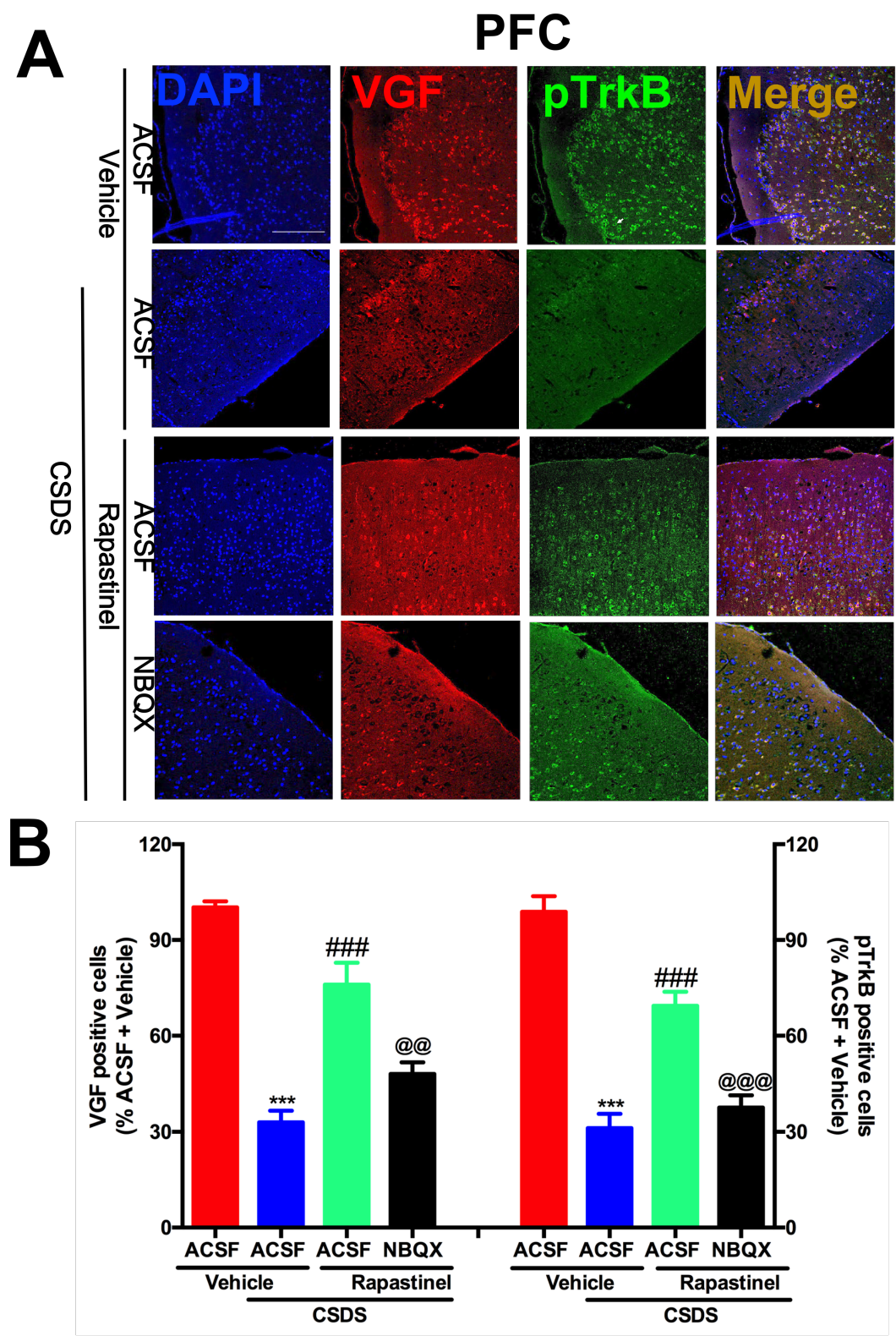
**

**Figure S2. Pretreatment with an AMPAR antagonist into PFC blocks rapastinel induced VGF release and activation of TrkB in mice.**

Given that the AMPAR activation-induced VGF release and TrkB activation involve in the rapid-acting antidepressant-like actions in mice. (A) We examined the expressions of VGF and pTrkB using the immunohistochemistry in the PFC of mice. (B) We found that single treatment with rapastinel significantly reversed the down-regulation of VGF and pTrkB induced by CSDS in the PFC of mice. However, the pretreatment with NBQX significantly remitted these effects of rapastinel in the PFC of mice. n= 5 per group. ***P< 0.001 vs. Vehicle + ACSF group; ###P < 0.001 vs. CSDS + Vehicle + ACSF group; @P < 0.05, @@P < 0.01, @@@P < 0.001 vs. CSDS + Rapastinel + ACSF group. All data are mean±SEM.

**Original gels of western blotting**


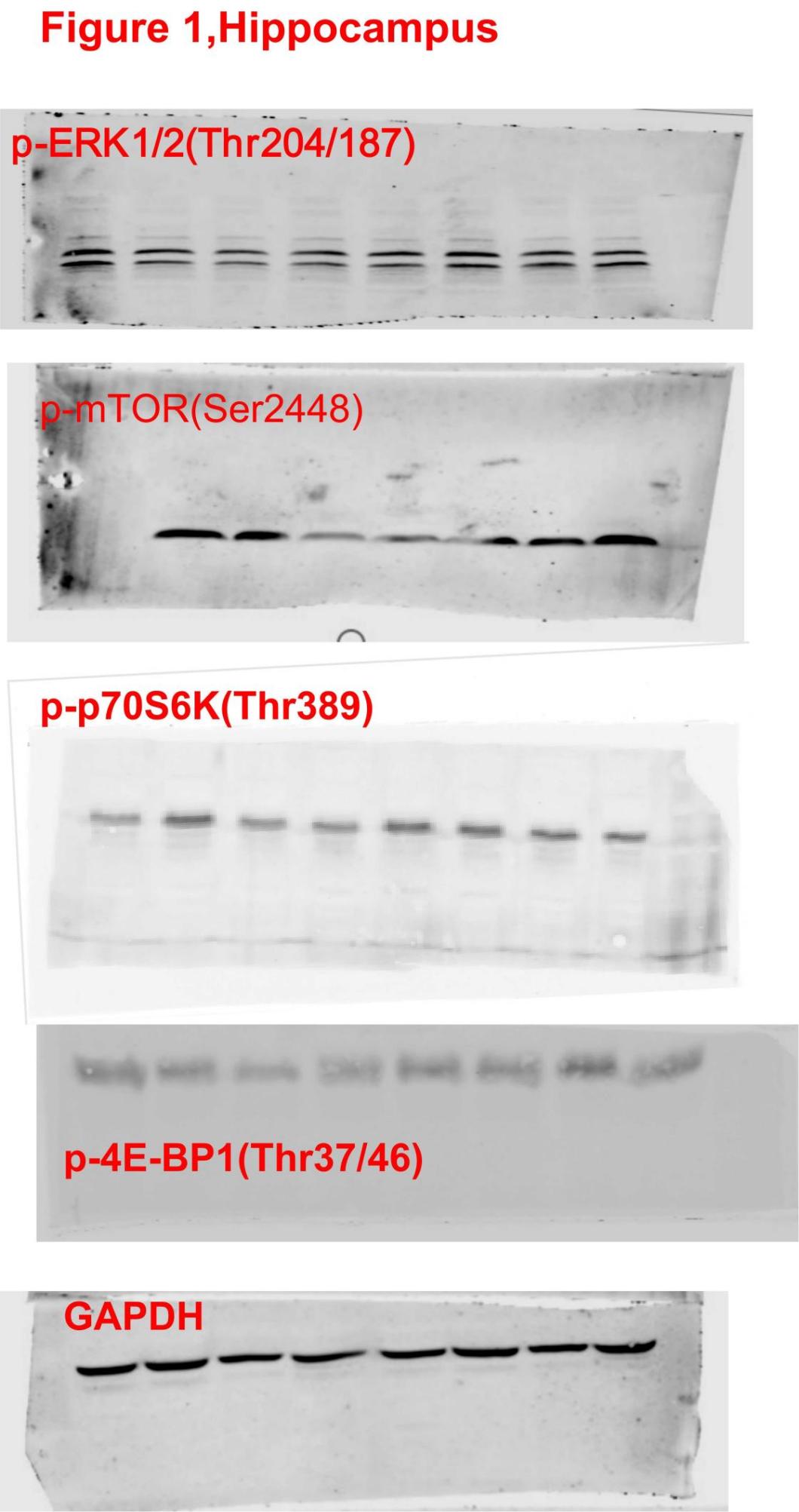


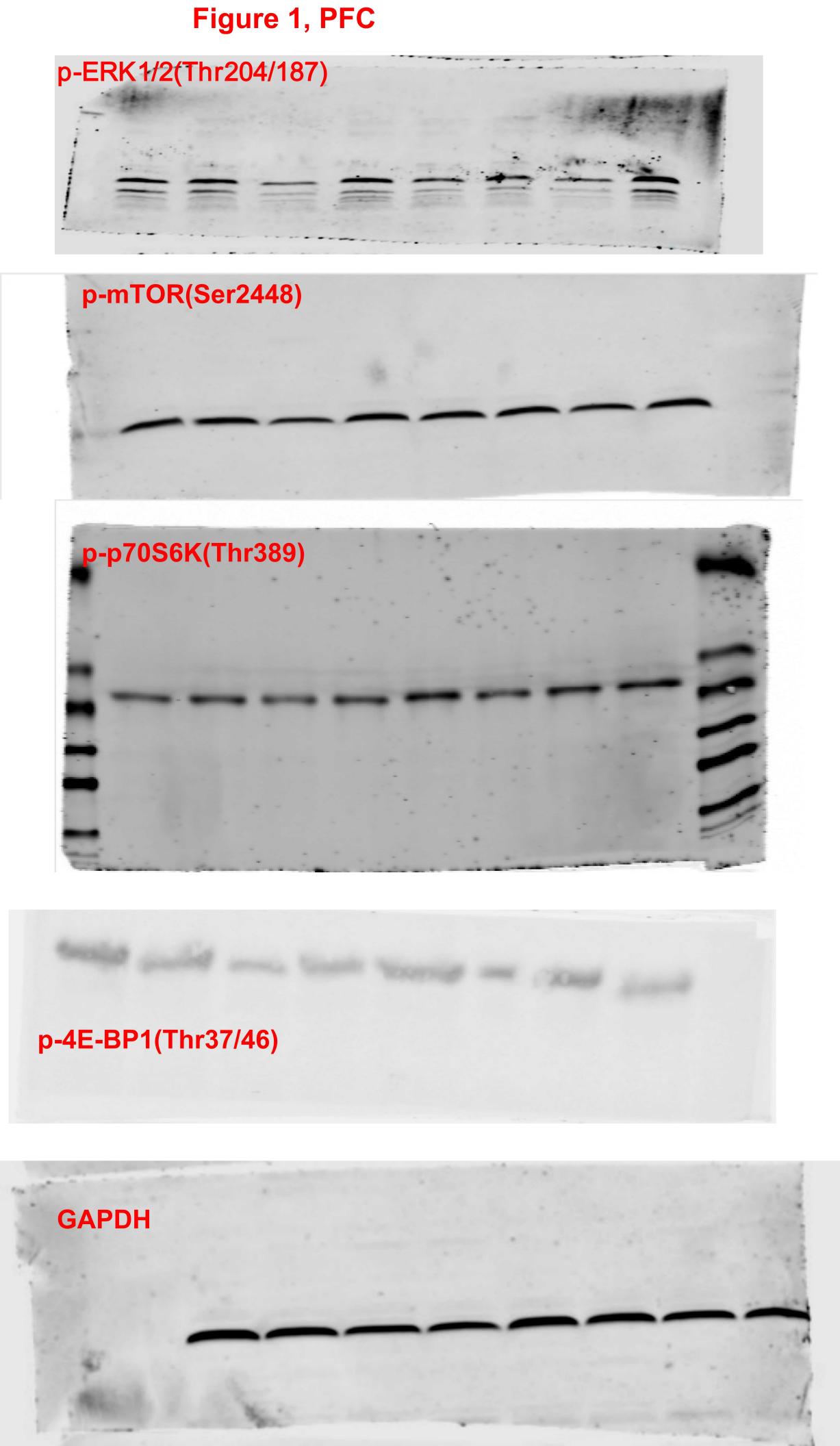


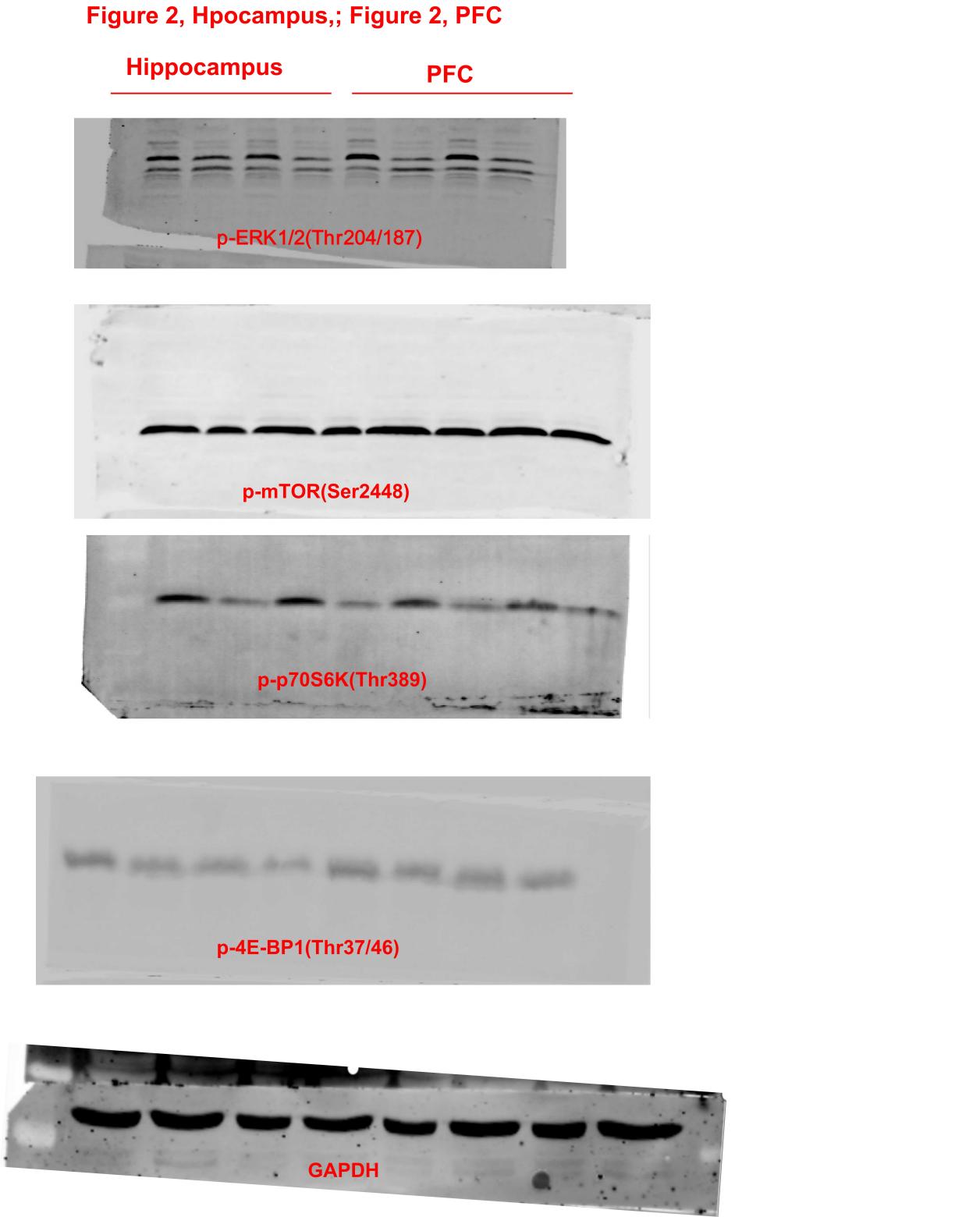


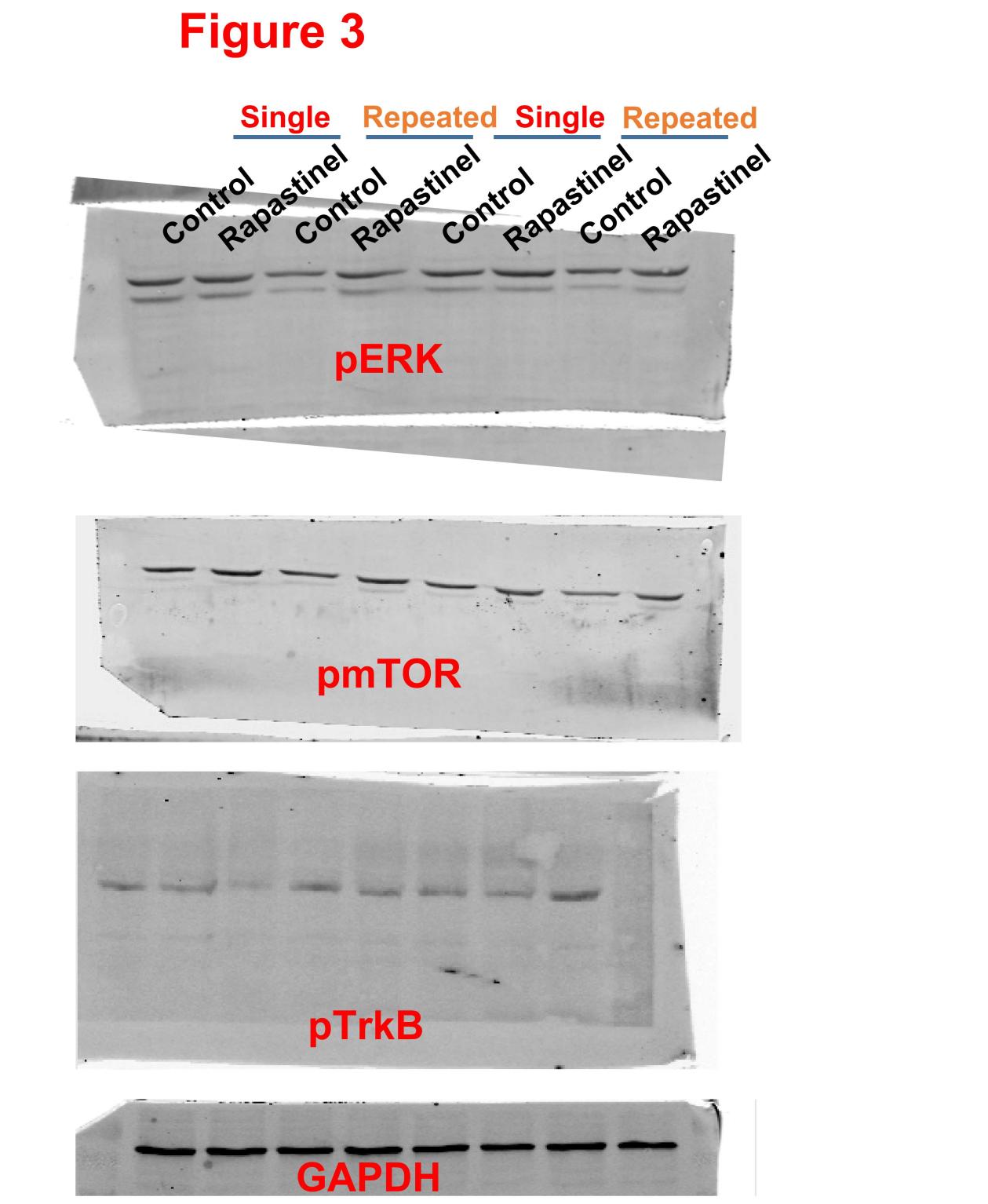

Supplement: Supplementary file 1 — Supplementary data [file 41398_2022_2290_MOESM1_ESM.docx]
